# Clinical Characteristics, Life Adversities and Personality Traits in Monozygotic Twins With, at Risk of and Without Affective Disorders

**DOI:** 10.3389/fpsyt.2018.00401

**Published:** 2018-08-31

**Authors:** Ninja M. Ottesen, Iselin Meluken, Thomas Scheike, Lars V. Kessing, Kamilla W. Miskowiak, Maj Vinberg

**Affiliations:** ^1^Copenhagen Affective Disorder Research Centre, Copenhagen University Hospital, Copenhagen, Denmark; ^2^Department of Biostatistics, University of Copenhagen, Copenhagen, Denmark; ^3^Institute of Psychology, University of Copenhagen, Copenhagen, Denmark

**Keywords:** affective symptoms, traits, childhood trauma, life events, twins, monozygotic

## Abstract

**Background:** Affective disorders have a long-term impact on psychiatric health and are caused by multiple interacting factors including familial risk, childhood adversity, life events and personality traits.

**Methods:** In this study, monozygotic twins (MZ) at familial risk (indexed by affective disorder in their co-twin; high-risk group), affected MZ twins (indexed by a diagnosis with affective disorder) and MZ twins with no family history of affective disorder (low-risk group) were identified through cross-linking of nation-wide Danish registers. In total, 204 MZ twins were included and psychopathology, personality traits and life adversity were evaluated by semi-structured interviews and questionnaires.

**Results:** Affected MZ twins presented with more subclinical affective symptoms and were functionally impaired as evidenced by higher unemployment rates and reduced functional status. The affected and the high-risk groups reported more childhood adversity and had experienced more stressful life events than the low-risk group. A direct comparison within the discordant twin pairs showed that the high-risk twins presented fewer affective symptoms, better functional status, more extraversion and lower neuroticism scores than their affected co-twins although they had equal levels of life adversity as their affected co-twins.

**Conclusion:** These findings add to the evidence indicating that patients experience higher neuroticism, persistent subclinical symptoms and reduced socio-occupational function after affective episodes. Additionally, neuroticism and extraversion seem capable of moderating the sensitivity to exposure from the environment.

## Introduction

Affective disorders are among the most disabling diseases worldwide (1) and there is a need for better identification of risk and resilience markers and improve early intervention. Affective disorders aggregate in families and are moderate to highly heritable; twin studies have consistently shown high heritability rates in bipolar disorder (0.36–0.80) and moderate rates in unipolar disorder (0.23–0.67) (2–5). However, studies confirming the heritability of affective disorders also demonstrate the existence of a large influence from environmental factors (6).

The landmark study by Kendler and colleagues (7) showed a joint effect of stressful life events (SLE) and genetic liability suggesting a genetic control on the depression inducing effects of SLE. Thus, monozygotic (MZ) twins at risk for affective disorders had the highest risk of onset of an affective episode after the experience of a major SLE within the last year (7). However, more distal events such as childhood adversity (e.g., familial death, parental abuse, sexual abuse) are also associated with profound long-term illness risk (8, 9). Gene-environment interactions reflect a causal mechanism where one genetic variant or one environmental factor contributes to the causation of a condition in the same individual with the genetic factor influencing the sensitivity to exposure from the environment (10) and thereby explain why individuals respond differently to environmental factors and are more prone to affective disorder after exposure to a SLE (11). As MZ twins share almost identical genes, studies including concordant and discordant MZ twins provide a particularly strong methodology for the study of risk markers (12, 13). High-risk studies offers an opportunity to study risk factors and provide insight into inherited vulnerability and causal pathways without the effects of potential changes associated with the burden of illness (14). Including identical twins in a high-risk design thus creates an innovative approach by using an “ultra high-risk” design in the study of risk and resilience factors, as the healthy identical twin have resisted the disorder onset irrespectively of their genetic predisposition. Therefore, they may express traits associated with resilience and/or compensatory mechanisms. Using this approach creates a unique opportunity to identify intermediate phenotypes (endophenotypes). By comparing unaffected high-risk individuals, with affected individuals and individuals without a predisposition for the disorder, it is possible to disentangle factors associated with resilience, vulnerability and also factors that may reflect scar effects of the disorder.

### Objectives

This study investigated risk factors for affective disorders using an “ultra-high-risk design” by including MZ twins. The aims were to first to investigate whether affective symptoms, functional status, childhood adversity, SLE and personality traits are associated with risk for affective disorders by comparing a cohort of MZ twin pairs discordant and concordant for affective disorder with MZ twin pairs with no history of affective disorders, and second to investigate in the discordant pairs, whether childhood adversity and personality traits significantly differ within the pairs. We hypothesized that affected twins would report more affective symptoms, lower functional status and higher neuroticism scores than high- and low-risk twins, and that they would have experienced more childhood adversity and SLE than the low-risk twins. Finally, personality traits were hypothesized to differ between the discordant twin pairs.

## Materials and methods

### Design

This study was a population-based high-risk study including MZ twins identified by crosslinking three Danish registers. First, *The Danish Twin Registry (DTR)* which includes more than 85,000 twin pairs and covers the Danish twin-cohorts since 1870 (15). Second, *the Danish Civil Registration System*, which was established in 1968 and provides all Danish citizens with a unique identification number (16). Finally, *The Danish Psychiatric Central Research Register* (DPCRR), which contains information on all admissions to psychiatric departments in Denmark since 1969, and since 1995 all outpatient contacts (17).

### Participants and recruitment

By cross-linking the DTR and the DPCRR, a cohort of MZ twin pairs was identified; these were classified into three groups (1) affected twins (twins in remission or partial remission with a affective disorder), (2) unaffected high-risk twins with a co-twin history of mood disorder, or (3) low-risk twins with no personal or family history of affective disorder. In the concordant twin pairs, both twins had at least one prior affective episode, while in the discordant twin pairs, one of the twins had an affective disorder (the affected twin) and the co-twin had no prior affective episodes (the discordant, high-risk twin). In the psychiatrically healthy twin pairs, both twins had no personal history or any first-degree relatives with affective disorders (low-risk twins).

Participants were included if, according to the register linkage, they had had a prior ICD-10 diagnosis of either a single depressive episode/recurrent depression (F32-33.9) or a single manic episode/bipolar disorder (F30-31.9, F 34.0, F38.0) between January 1, 1995 and June 1, 2014 and if the diagnosis was confirmed in a subsequent face to face Schedules for Clinical Assessment in Neuropsychiatry (SCAN) interview (18). All participants were in remission or partial remission on the day of investigation with the Hamilton Depression Rating Scale-17 items (HDRS-17) (19) and the Young Mania Rating Scale (YMRS) (20). Exclusion criteria include prior head trauma with unconsciousness and sequelae, birth weight < 1,300 g, pregnancy, current substance abuse, severe somatic illness, HDRS-17 or YMRS > 14 or if they were dizygotic. Additionally, the low-risk twins were excluded if they had a first-degree relative with an organic mental disorder, schizophrenia spectrum disorders or affective disorders.

Recruitment took place from December 2014 until January 2017. The participants were invited by letter and if there was no response from the participant, another remind letter was posted, and then we attempted contacted via phone. If they declined to participate, they were asked to answer a brief questionnaire. At the assessment, fasting blood- and urine samples were collected between 9 and 11 a.m. and ratings and a SCAN interview ware conducted by two PhD students (IM and NMO, blinded to the DPCRR register diagnoses). Questionnaires were completed during the day (preferable), or returned by letter.

The study was approved by the Danish National Board of Health (Sundhedsstyrelsen), the data protection agency (2014-331-0751) and the local ethical committee (H-3-2014-003). The project was competed in accordance with the Helsinki-Declaration-2 and all participants gave written informed consent.

### Measures

Functional status was assessed using the Functioning Assessment Short Task (FAST), which include 24 items divided into six areas: autonomy, occupational functioning, cognitive functioning, financial issues, interpersonal relationships and leisure time. Scores above 11 indicate functional impairment. The FAST is validated to evaluate functioning in a bipolar population (21, 22).

Self-rating of childhood trauma was assessed using the Childhood Trauma Questionnaire (CTQ), which is a 28-item scale with good reliability and validity (23). The questions include a range of experiences in childhood and adolescence, classified into categories: physical, sexual, and emotional abuse, and physical and emotional neglect. In the Stressful Life Events (SLE) questionnaire the participants were asked about recent SLE in the year prior to the interview: nine “personal” events concerning marital problems, illness, work problems, assaults, robbery, divorce etc. which may have happened to themselves and 22 “network” which may have happened to their co-twin. Finally, the questionnaire also includes 10 questions assessing the experience of SLE lifetime before. A Danish version translated with permission from the author, was used (24).

Personality traits were assessed using a Danish version of the Eysenck Personality Questionnaire (EPQ) (25). The categories for extraversion and neuroticism have been validated and found to be reliable (26). Screening for comorbid personality disorder was performed using the interviewer-administered Standardized Assessment of Personality–Abbreviated Scale (SAPAS). The SAPAS is a short version of the Standardized Assessment of Personality (27), and is an eight-item interview used to screen for comorbid personality disorder. The maximum score is 8, with a cut-off score ≥3 (28). The Danish version and the cut-off score have been validated in a Danish sample (29).

### Statistical analyses

Overall, continuous dependant variables were analyzed with mixed model analysis of variance where the intra twin-pair dependence was accounted for by using twin pair identification numbers as random factors. Categorical dependant variables were analyzed with logistic regression models and the intra twin-pair dependence was done by use of the generalized estimating equations (GEE) model for twin pairs. In all models, group was considered the fixed factor. Spearman correlations were used to test the associations between childhood trauma, personality traits, HAMD-17 and FAST. Analyses were conducted using the mixed, genmod and glimmix procedures in SAS 9.4 (SAS Institute Inc.).

Our analyse strategy were 3-fold, first we compared the following three groups (1) remitted or partially remitted MZ twins with a personal history of unipolar or bipolar disorder (affected), (2) unaffected MZ twins with a co-twin history of unipolar or bipolar disorder (high-risk), and (3) MZ twins with no personal or first-degree family history of unipolar or bipolar disorder (low-risk). In these primary analyses, we wanted to test whether the affected twins reported more affective symptoms, lower functional status and higher neuroticism scores than high- and low-risk twins and whether they had experienced more childhood adversity and SLE than the high-risk and low-risk twins. To account for the dependence between a twin-pair, the twin-pair identification number was treated as a random factor. If any statistically significant associations were found, *post-hoc* pair-wise analyses were performed between the three groups, aiming to identify the exact group difference.

In the secondary analyses (concordance analyses), we repeated the analyses at twin pair level and studied whether the concordant twin pairs (with a presumed higher genetic load than discordant pairs) would express poorer outcome than the discordant twin pairs. The genetic risk was investigated by comparing the following three groups: (1) the concordant affected twin pairs (both twins affected), (2) the discordant twin pairs (one twin affected, the other twin healthy) and (3) low-risk twin pairs (both twins healthy). These analyses were performed in a similar manner to the primary analyses.

Finally, in the tertiary analyses we wanted to elucidate whether the risk factors separated between the discordant twin pairs. Thus, the within pair difference between the affected and the unaffected twins in the discordant twin pairs was investigated using paired *t*-tests (discordant analyses). Simple main effect analyses were used to decompose significant interactions. Comparisons in between the affected individuals in the concordant and discordant twin pairs, respectively were done using one sample *t*-test.

## Results

As seen in Table 1, the register linkage (June 1st, 2014) identified 307 MZ twin pairs aged between 18 and 65 years, who were born between 1949 and 1996 and where both twins were alive and one or both twins had had a affective disorder diagnosis according to ICD 10 (F30-F39), being either concordant or discordant for unipolar and bipolar disorder, borne between 1949 and 1996. Of the 307 twin pairs; 35 pairs (11%) were concordant for affective disorder, 23 pairs for unipolar disorder and 12 pairs for bipolar disorder. Only participant aged 18–50 years were invited, to participate in the study leaving 238 eligible twin pairs (right column, Table 1).

**Table 1:** Number of monozygotic twins concordant and discordant for unipolar and bipolar disorder.

	**Unipolar borne 1949–1996**	**Bipolar *N* = 307**	**Unipolar borne 1964–1996**	**Bipolar *N* = 238**
Concordant	23 (9%)	12 (25%)	20 (10%)	8 (23%)
Discordant	236 (91%)	36 (75%)	183 (90%)	27 (77%)
In total	259	48	203	35

*Results from the register linkage between the Danish Twin Registry and the Danish Central Psychiatric Research Registry*.

### Participants (Figure 1)

In total, 408 monozygotic twins (204 twin pairs) were invited and 44 were excluded. A total of 155 MZ twins either declined to participate (*n* = 101) or were not able to be reached (*n* = 54). Therefore, 209 MZ twins were included in the study (57.4%). Five were subsequently excluded due to a personal or first-degree family history of schizophrenia or schizotypal disorder. The final analyses included 204 twins where 115 participants had a diagnosis of affective disorder, 49 were at high-risk and 40 were at low-risk. In the secondary concordance analyses, only twin pairs where both twins participated were included (*n* = 89 pairs) of these 25 MZ twin pairs were concordant and 45 MZ twin pairs were discordant for affective disorder and 19 MZ twin pairs were included as controls. Finally, for the discordant intra-pair analyses, data from the 45 discordant MZ twin pairs were included.

**Figure 1:**
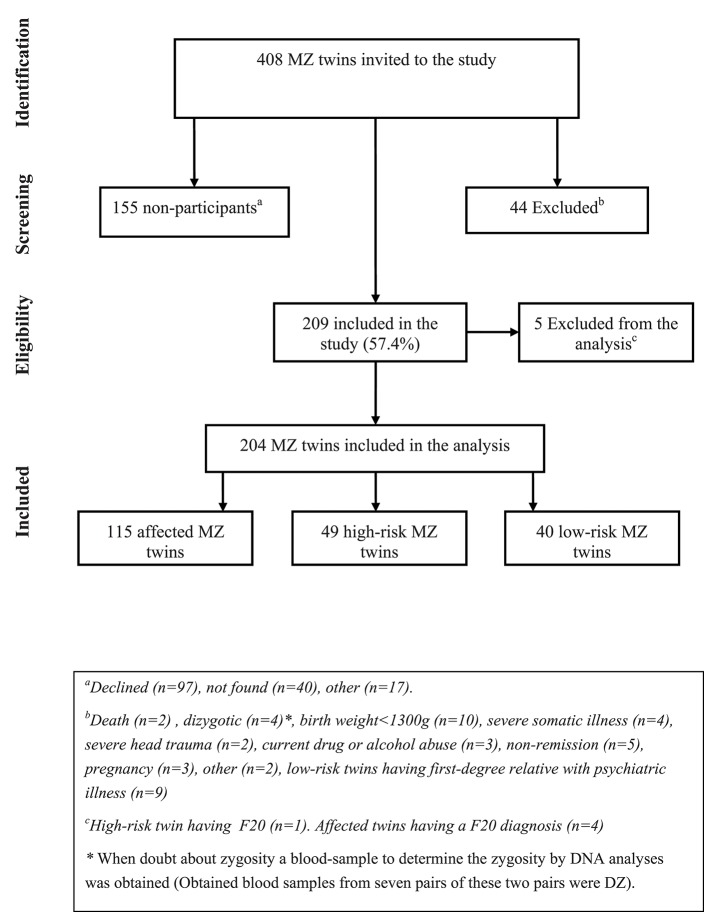
Flow charge, participants monozygotic (MZ) twins having an affective disorder diagnosis (affected twins), or a co-twin with affective disorder (high-risk twins) or no family history of mood disorder (low-risk twins).

### Participants vs. non-participants (Table 2)

Of the eligible participants, 155 (42%) did not participate. Of these, it was not possible to track 40 (26%) individuals. Concerning all non-participants, data were drawn from the registers and there were no significant differences in terms of age, sex, geography, or diagnoses. Concerning, the non-participants, the 26% who did not respond more often had a diagnosis (62.5%) than those who responded, but declined (46%; *p* = 0.05).

**Table 2:** Characteristics of participants vs. non-participants, confidence interval (CI).

**Number**	**Participants 209**	**Non-participants 155**	***P***
Age, years (mean, CI)	36.6 (35.4–37.7)	37.3 (36.0–38.6)	0.04
Sex, female, *N* (%)	153 (71)	110 (71)	0.87
Diagnose^*^, *N* (%)	123 (58)	78 (50)	0.15
Geographic^**^ (%)	130 (60)	91 (59)	0.25

* *Had a diagnose from ICD-10 according to the Danish Psychiatric Central Research Register*.

** *Living in eastern Denmark, near the research center*.

### The cohort

As seen in Table 3, the three groups (1) concordant and discordant affected twins, (2) discordant twins, and (3) concordant unaffected MZ twins) were comparable in terms of on age, sex, years of education, and civil status. However, the affected group was significantly less often employed or studying than the high- and low-risk groups, and the affected group had significantly more comorbid non-affective diagnoses. In the affected group, 61% were treated with medication at the time of inclusion (Table 4) and one high-risk twin received an antidepressant with anxiety as the indication.

**Table 3:** Risk status and socio-demographic variables; affected, high-risk and low-risk monozygotic twins.

**Risk status**	**Affected**	**High-risk**	**Low-risk**	***P***
Number	115	49	40	
Age years (mean, CI)	35.9 (34.2–37.6)	36.7 (34.1–39.3)	35.8 (31.5–40.1)	0.86
Gender *N* (%)				
Female	83 (70.3)	33 (67.4)	32 (80.0)	0.22
Years of education: estimate mean (CI)	14.4 (13.8–15.0)	15.6 (14.7–16.5)	14.8 (13.3–16.3)	0.06
Civil status, *N* (%):				
Married/in relationship	76 (66.0)	37 (75.5)	29 (72.5)	0.32
Civil (home) status, *N* (%), Living alone	42 (36.5)	10 (20.4)	10 (25.0)	0.08
Children: *N* (%),				
No children	55 (47.5)	17 (34.7)	15 (37.5)	0.15
In occupation *N* (%):				
(Employment + education)	81 (68.4)	45 (91.8)	38 (95.0)	0.0001

**Table 4:** Diagnosis and medication status; affected, high-risk and low-risk monozygotic twin (*N* = numbers).

**Risk status**	**Affected**	**High-risk**	**Low-risk**	***P***
**DIAGNOSIS** ***N*** **(%)**				
Bipolar disorder	31 (27)	0 (0)	0 (0)	
Unipolar disorder	83 (72)	0 (0)	0 (0)	
Other non-affective disorder, *N* (%)	61 (53.4)	12 (24.5)	6 (15)	0.0001
Age of onset, years	23.0	NA	NA	
N affective episodes (mean)	5.1	0 (0)	0 (0)	
N admissions (mean)	5.8	0 (0)	0 (0)	
**FAMILIAR DISPOSITION**, ***N*** **(%)**				
1. Degree relatives with affective disorder:	70 (61.4)	42 (87.5)	0 (0)	
2. Degree relatives with affective disorder:	54 (47.3)	19 (39.6)	7 (17.5)	
Current medication, *N* (%)	70 (61)	1 (2)	0 (0)	
Antidepressiv	50 (71)	1 (2)	0 (0)	
Antipsychotic	18 (26)	0 (0)	0 (0)	
Mood stabilizer	22 (31)	0 (0)	0 (0)	

### Comparison of affective symptoms

As seen in Table 5, there were significant differences between the three groups in the *primary* analysis with regard to HDRS-17 scores (*p* < 0.0001). The affected group showed more subclinical symptoms with the high-risk group showing intermediate levels and the low-risk group showing the least symptoms. The *post-hoc* pair-wise group analysis revealed that the affected twins had significantly more subclinical affective symptoms than the high-risk group (*p* = 0.0002) and the low-risk group (*p* < 0.0001). The high -and low-risk groups did not differ significantly (*p* = 0.17).

**Table 5:** Hamilton depression rating scale-17 items (HDRS-17), Young Mania rating scale (YMRS), functional assessment short task (FAST), recent stressful life events (SLE) and life time before, childhood adversity and personality traits according to Eysenck personality questionnaire (EPQ) and standardized assessment of personality–abbreviated scale (SAPAS) in affected, high-risk and low-risk monozygotic twins, in brackets confidence interval (CI).

**Primary analyses**						***Post-hoc*** **analyses** ***p*****-values**		
**Risk status, mean (CI)**	**Affected (AF)**	**High-risk (HR)**	**Low-risk (LR)**	***P***	***P* adjusted f HDRS-17**	**AF vs. HR**	**AF vs. LR**	**LR vs. HR**
HDRS-17	4.8 (4.2–5.4)	2.8 (1.9–3.7)	1.9 (0.9–2.8)	< 0.0001		0.0002	< 0.0001	0.17
YMRS	1.8 (1.5–2.2)	1.6 (1.0–2.1)	1.3 (0.7–1.8)	0.2	0.9	^**^	^**^	^**^
**FAST**								
Autonomi	0.8 (0.6–1.2)	0.3 (−0.0–0.7)	0.2 (−0.2–0.6)	0.008	0.2	0.02	0.01	0.62
Cognition	3.0 (2.6–3.4)	1.2 (0.5–1.8)	1.3 (0.6–2.0)	0.0001	0.001	< 0.0001	< 0.0001	0.78
Economy	0.4 (0.3–0.6)	0.2 (−0.1–0.4)	0.2 (−0.1–0.4)	0.09	0.43	^**^	^**^	^**^
Social	2.7 (2.2–3.2)	0.9 (0.3–1.s7)	0.9 (0.2–1.7)	0.0001	0.06	0.0001	0.0003	0.96
Leisure	0.9 (0.7–1.1)	0.5 (0.2–0.8)	0.3 (−0.2–0.7)	0.008	0.22	0.07	0.01	0.37
Total	13.4 (11.4–15.7)	3.3 (0.7–5.9)	3.4 (0.6–6.3)	0.001	0.0001	< 0.0001	< 0.0001	0.97
**SLE**								
Interpersonal^a^	1.1 (0.8–1.3)	0.8 (0.5–1.2)	0.6 (−0.0–1.3)	0.30	0.69	^**^	^**^	^**^
Alcohol^a^	0.4 (0.3–0.6)	0.6 (0.4–0.7)	0.4 (0.1–0.4)	0.10	0.14	^**^	^**^	^**^
Other^a^	1.1 (0.9–1.3)	1.1 (0.7–1.4)	0.5 (0.1–0.9)	0.02	0.07	0.84	0.006	0.88
Lifetime before^b^	2.8 (2.4–3.1)	2.4 (1.8–2.9)	1.9 (1.3–2.5)	0.02	0.22	0.26	0.01	0.22
**CHILDHOOD ADVERSITY**								
Physical abuse	5.7 (4.4–6.1)	5.9 (5.3–6.4)	5.3 (4.7–5.8)	0.24	0.34	^**^	^**^	^**^
Emotional abuse	9.3 (8.5–10.1)	8.9 (7.6–10.2)	6.6 (5.3–7.9)	0.002	0.01	0.56	0.0005	0.01
Sexual abuse	4.7 (4.4–5.1)	4.3 (3.8–4.8)	3.6 (3.1–4.1)	0.001	0.002	0.16	0.001	0.06
Emotional neglect	11.2 (10.1–11.2)	10.7 (9.2–12.2)	7.6 (6.1–9.1)	0.0004	0.004	0.58	< 0.0001	0.001
Physical neglect	7.4 (6.9–8.0)	7.0 (6.1–7.9)	5.7 (4.8–6.6)	0.008	0.05	0.42	0.002	0.05
Deceiving	9.2 (8.6–9.8)	10.0 (9.1–10.9)	11.6(10.7–12.6)	0.0001	0.05	0.14	< 0.0001	0.02
Total	38.0 (35.8–40.2)	36.8 (33.3–40.3)	28.7 (25.2–32.3)	0.0001	0.001	0.58	< 0.0001	0.002
**EPQ**								
Extraversion	11.6 (10.6–12.6)	13.4 (11.9–15.0)	15.7 (12.5–18.9)	0.01	0.05	0.03	0.01	0.15
Neuroticism	11.8 (10.8–12.8)	7.4 (5.9–8.9)	6.4 (4.7–8.0)	0.0001	0.0001	0.0001	< 0.0001	0.37
L–Lie	9.6 (8.4–10.3)	10.0 (8.9–11.2)	10.8 (9.6–12.0)	0.22	0.30	^**^	^**^	^**^
Psychoticism	3.7 (3.3–4.1)	3.0(2.4–3.6)	3.1 (2.4–3.7)	0.08	0.25	^**^	^**^	^**^
SAPAS	2.7 (2.4–3.0)	2.1 (1.7–2.6)	1.9 (1.5–2.4)	0.007		0.03	0.01	0.50

** *No statistically significant differences*,

a Life events in the year prior to the interview and

b *life events in their lifetime before the interview*.

In the *secondary* concordance analysis, the concordant twin pairs had the highest HDRS-17 scores and the low-risk twins the lowest scores, these differences were all statistically significant (*p* = 0.0001).

Finally, in the *tertiary* discordance analyses, the affected twins in the discordant twin pairs had significantly more depressive symptoms than the unaffected twins (*p* = 0.01).

### The functioning assessment short task

In the *primary* analyses, the affected group had higher scores (indicating more impairment) on the FAST than the high-risk twins (*p* < 0.0001) and the low-risk twins (*p* < 0.0001) on the total score and on almost all subscale scores—except the subscale Economy (see Table 5), and for the subscales Autonomy and Leisure time, the differences were only significant between the affected and the low-risk twins. These between-group differences remained significant for the FAST total score (*p* = 0.001) and the cognition subscale score (*p* = 0.001) also after adjustment for HDRS-17 scores.

In the *secondary* analyses, the concordant twin pairs showed significantly higher FAST scores in comparison with the low-risk twin pairs in most subcategories, except “autonomy” and “economy.” Regarding the subscale social and FAST total, the differences were also significant between the concordant and discordant twin-pairs.

Finally, in the *tertiary* discordance analysis, the affected twins exhibited significantly poorer functioning than the unaffected twins in all categories except the leisure subscale (*p* < 0.0001).

### Childhood adversity and stressful life events

In the *primary* analyses as can be seen in Table 5, both the affected twins and the high-risk twins, on average, experienced significantly more childhood adversity, than the low-risk twins on almost all subscales: emotional abuse, affected vs. low-risk twins (*p* = 0.0005), high-risk vs. low-risk twins (*p* = 0.01), sexual abuse, affected vs. low-risk twins (*p* = 0.001), comparing high-risk vs. low-risk twins the difference was reduced to a strong trend (*p* = 0.06). Emotional neglect, affected vs. low-risk twins (*p* = 0.0001), high-risk vs. low-risk twins (*p* = 0.01), physical neglect, affected vs. low-risk twins (*p* < 0.0001) high-risk vs. low-risk twins (*p* = 0.05), deceiving, affected vs. low-risk twins (*p* < 0.0002), high-risk vs. low-risk twins (*p* = 0.02) and childhood trauma total, affected vs. low-risk twins (*p* = 0.0001), high-risk vs. low-risk twins (*p* = 0.002). The above-described results remained statistically significant after adjusting for HDRS-17 score. The low-risk twins had experienced significantly fewer SLE during the last 12 months than the affected twins, in the subcategories other (*p* = 0.006) and lifetime before (*p* = 0.01); although, the group differences were only borderline significant when adjusted for the HDRS-17 score (*p* = 0.07) and not statistical significant for lifetime before (*p* = 0.21).

In the *secondary* analyses the low-risk twins had experienced significantly fewer childhood adversities than the concordant (*p* = 0.001) and discordant twin pairs (*p* = 0.0002), and this also applied to the experience of sexual abuse during childhood. The concordant twin pairs had experienced significantly more SLE during the last 12 months as compared to the low-risk twins, in all subcategories. However, only the subcategory “other” remained statistically significant when adjusted for HDRS-17 score. Regarding childhood trauma, the results remained statistically significant when adjusted for the HDRS-17 score.

Finally, in the *tertiary* discordance analyses, no differences were found between the affected and the unaffected twin in the discordant twin pairs in regard to childhood adversity and SLE.

### Personality traits and the standardized assessment of personality–abbreviated scale (SAPAS)

As seen in Table 5, the affected group exhibited significantly higher scores on neuroticism than the high-risk group (*p* = 0.0001), and the low-risk group (*p* < 0.0001) and lower scores on extraversion than the high-risk (*p* = 0.03) and the low-risk group (*p* = 0.01), even after adjusting for HDRS-17 score in the *primary* analyses. Using the SAPAS, as a measure of risk of having a comorbid personality disorder, the affected twins had higher scores than the high-risk twins (*p* = 0.03) and the low-risk twins (*p* = 0.01). However, none of the groups had mean scores beyond the SAPAS cut-off score ≥3.

The *secondary* analyses were consistent with the primary analyses, the concordant twin pairs had significantly higher neuroticism scores than the discordant twin pairs (*p* = 0.002) and the low-risk twin pairs (*p* = 0.0001) and higher extraversion scores than the low-risk twin pairs (*p* = 0.02). Further, the discordant twin pairs also exhibited higher neuroticism scores than the low-risk twin pairs (*p* = 0.04). When adjusted for HDRS-17 score, the difference disappeared for the category extraversion (*p* = 0.87), but remained for neuroticism (*p* = 0.03). For the SAPAS, there were no group differences in the concordance analyses.

Finally, in the *tertiary* discordant analysis, the affected twins in the discordant twin pairs had significantly higher neuroticism scores (*p* = 0.0002) and higher SAPAS scores (*p* = 0.04) than the unaffected twins.

### *Post-hoc* analyses

To elucidate whether there were any differences between individuals or twin pairs with unipolar vs. bipolar disorder, additional analysis were conducted to compare these two groups. We found that twins having bipolar disorder had lower overall functioning (mean UD = 11.8, mean BD = 17.7, *p* = 0.02), they had experienced more LE lifetime before (mean UD = 2.48, mean BD = 3.48, *p* = 0.02) and they reported significantly higher extraversion scores (mean UD = 10.6, mean BD = 13.02, *p* = 0.04) and higher psychotism scores(mean UD = 3.43, mean BD = 4.57, *p* = 0.02). However, there were no significant differences between UD and BD twins in neuroticism scores. Further when comparing the high-risk twins predisposed for UD (*N* = 37) and BD (*N* = 12), no statistically significant differences in between the two groups were found (results not presented).

As the affected concordant twins (*N* = 70) may differ from the affected discordant twins (*N* = 45) comparisons between all measures in between these two groups were conducted and no significantly statistical differences were found (result not presented).

Exploratory analyses of the associations between personality traits, childhood trauma, and clinical characteristics across the whole sample revealed positive associations between neuroticism and the total number of childhood trauma (*r* = 0.35, *p* = 0.0001), FAST total score (*r* = 0.51, *p* = 0.0001) and HDRS-17 score (*r* = 0.23, *p* = 0.001), indicating that participants with high neuroticism scores had experienced more childhood adversity, showed greater functional impairment and had more subsyndromal affective symptoms. However, there was no association between extroversion scores and childhood trauma (*r* = −0.11, *p* = 0.15) or HDRS-17 scores (*r* = −0.06, *p* = 0.42). There was a statistically significant negative association between extroversion and FAST total score (*r* = −0.39, *p* = 0.0001), indicating that participants with higher extroversion scores reported an overall higher functional status. Finally, there were statistically significant positive associations between HDRS-17 score and childhood trauma (*r* = 0.20, *p* = 0.007) and FAST total score (*r* = 0.29, *p* = 0.0001).

## Discussion

This until now largest MZ high-risk study in affective disorder revealed, in accordance with our hypothesis, that in comparison with healthy high-risk and low-risk twins, affected MZ twins had more subclinical affective symptoms, higher neuroticism scores, lower extraversion scores and they were more often unemployed and experienced more functional impairment. Together, these findings point to a possible scar effect of the affective disorder as the high-risk twins did not differ from the low-risk twins. Further the affected group and the high-risk group had experienced more childhood adversity and SLE than the low-risk twins. Notably, the high-risk group also reported significantly more childhood adversity than the low-risk twins. Further examination of the discordant MZ twin pairs revealed no differences between the twins in terms of childhood adversity and SLE. Finally, as hypothesized, the affected twins in the discordant twin pairs had higher neuroticism and extraversion scores and higher scores on the screening tool for personality disorder, as compared to the unaffected twins. As the affected twins and their healthy co-twins reported equal levels of childhood and lifetime adversity, it appears likely that personality traits impact the sensitivity to environmental adversity.

### Subclinical depressive symptoms, functional status and employment

The lower functional impairment, more unemployment and increased depressive symptoms among the affected twins with previous affective episodes than high-risk and low-risk are consistent with prior studies (30–32). We have previously found that subclinical depressive symptoms were increased in high-risk twins compared with low-risk twins (12), a finding that was not replicated in the present study, although, the high-risk group numerically had higher scores. This may be due to decreased statistical power due to the lower number of high-risk twins included in the present study. Nevertheless, our prior study did include high-risk twins only and not affected twins. Another study found an association between depressive symptoms and self-rated functional impairment among patients with affective disorders (32). In the present study, the assessment of functional impairment was interviewer-administered but still relied on the participant's subjective view of their everyday functioning.

### Childhood trauma and stressful life events

Several studies have shown that childhood adversity is associated with an increased risk of affective disorders in adult life (8, 33–35). Further, childhood sexual abuse in women extensively increases the risk for developing a wide range of psychopathology (8, 33), a finding recently replicated in a longitudinal twin study where childhood maltreatment was shown to predict early adult psychopathology (36). In the present study, a broad range of childhood trauma was investigated and we found that both affected twins and high-risk twins had experienced significantly more childhood adversity including emotional abuse, sexual abuse, emotional and deceiving in comparison with the low-risk twins. This suggests that childhood adversity thus seems to be a potent risk factor for the development of affective disorders. However, the present high-risk twins had not developed psychiatric disorders, which may be due to more “resilient” personality traits (lower neuroticism and higher extraversion). All twin pairs, except one pair, were raised together so they most likely had experienced the same childhood adversities, in line with our finding of no differences between the twins in discordant twin pairs.

The experience of recent SLE increases the risk of affective disorders (7, 24, 37, 38). Here, both the affected twins and the high-risk twins reported more SLE in the recent 12 months as compared to low-risk twins. Notably, the group differences were reduced to a trend level when adjusted for HDRS-17 score indicating that subclinical symptoms may influence the subjective experience of SLE and probably also the subjective impact of SLE. Further, neuroticism and SLE seem to have additive effects and increase the overall risk of affective symptomatology (39, 40).

### Personality traits and the standardized assessment of personality–abbreviated scale

The personality trait neuroticism has been associated with onset of affective disorders in previous studies (13, 41, 42). Neuroticism is also suggested to be a mediating factor for the relation between childhood adversities on adulthood depression (38). In the current study, the affected twins in the discordant twin pairs had higher neuroticism scores; this is in accordance with the scar hypothesis whereby the experience of affective disorder episodes has long lasting effects leading to a scar effect on personality causing personality changes (43). However, looking at the genetic origins of neuroticism, a recent genome-wide study identified a strong genetic correlation between neuroticism and major depressive disorder but not bipolar disorder (44). Major depression and neuroticism have substantial overlaps and the before mentioned study by Kendler et al. also showed that approximately 55% of the genetic liability of MDD was shared with neuroticism (45). This is in line with the later findings from a meta-analysis of genome-wide association studies for neuroticism and the association with major depression (46). Twin studies suggest that approximately 40% of the trait variance for neuroticism is heritable and neuroticism in adulthood also seem stable over time (47, 48), speaking against an environmental transmission of individual differences in neuroticism. Nevertheless, in the present study we did find significant differences in neuroticism scores between the discordant twin pairs as the affected twins had higher scores than the non-affected twins pointing at two possibilities. First, that neuroticism may reflect trait vulnerability and the affected twins might thereby have been more vulnerable to environmental effect as childhood adversities. Second, although neuroticism is presumed to be stable over time (in adulthood) it could be a scar effect of either the early manifestations of the disorder or an early scar effect of childhood adversities.

Further, the trait of extraversion, is characterized by being outgoing, talkative, high on positive affect and having a need for external stimulation: this is associated with an overall beneficial impact on psychiatric health (49). Thus, the finding that high-risk twins had higher extraversion score than affected twins, and that this trait also distinguished between the discordant twin pairs emphasize extraversion as a potential resilience trait.

Comorbid personality disorders are common in affective disorders (50, 51) and are associated with poorer prognosis (52). The affected twin group exhibited higher scores than the other groups on the SAPAS, pointing to an increased risk for having a personality disorder among this group. However, the groups did not exhibit scores beyond the cut-off score, and the findings should be interpreted with caution, as the SAPAS questionnaire is a rather simplistic way of screening for personality disorders and does not discriminate between different types of personality disorders. Nevertheless, these finding indicates that both higher neuroticism and risk of comorbid personality disorder may be a consequence of previous affective episodes.

### Strengths and limitations

The large sample size of MZ twins, the comprehensive collection of data and the cross-linking of the Danish registers to identify the participants (which is a unique way to reduce selection bias) are all strengths of the current study. However, a consequence of including MZ twins only is that we included relatively few high-risk twins which may have resulted in decreased statistical power to detect differences between high and low-risk twins as suggested by the numerically, but not statistically significant, differences on many of the investigated measures (Table 5). In the present high-risk study design, it was possible to detect the effects of the disorder by comparing the affected twins with the high-risk twins and thereby detect the scar effects of the disorder and possible vulnerability traits. However, it was not possible to distinguish between genes and environment, as we did not include an additional cohort of dizygotic twins as done in a classical twin study. Finally, the cross-sectional design limits the possibility to draw causal conclusions; these may follow from a planned prospective follow up of this study. Given the cross-sectional study design, it is not possible to determine the predictive value of the present risk markers. However, we plan to examine this in a future follow-up study of the present cohort. Nevertheless, severe life adversities and subsyndromal clinical depressive symptoms seem to contribute substantially to the risk for later development of affective disorders based on previous findings from our group (13).

### Generalizability

It has been discussed that the low birth weight of twins could cause a higher risk of developing both physical and psychiatric disorders, as compared to singletons. However, large studies have emphasized that MZ twins do not differ from singletons in the frequency of diabetes, cancer, coronary heart disease, height or education level (53–55) and another study also found equal rates of bipolar disorder between MZ twins and singletons (56). These findings indicate that the twin cohort in this study is representative of the general population.

## Conclusion

Overall, the findings of this study add to the evidence suggesting that patients experience higher neuroticism, persistent affective symptoms, and reduced socio-occupational function after partial remission from affective episodes emphasizing that affective episodes leave behind a scar effect. This study thus highlights being diagnosed with an affective disorder has tremendous impact. The finding that unaffected twins from discordant twin pairs display higher extraversion and lower neuroticism, despite equal degrees of lifetime adversity suggest that personality traits are capable of moderating the sensitivity to exposure from the environment.

## Author contributions

MV and KM conceived and designed study. LK contributed to the conception and design. MV and KM obtained the funding. MV applied for the Data and the Ethical permissions and cooperated on the register linkage with the Danish Twin Registry. IM and NO recruited the patients and runned the study together with MV. NO and TS undertook the data extraction and the statistical analyses. NO and MV drafted the manuscript drafts and MV revised the final version. All authors had substantial contributions to the design, analysis, and interpretation, and participated in manuscript drafting or revisions.

### Conflict of interest statement

The authors declare that the research was conducted in the absence of any commercial or financial relationships that could be construed as a potential conflict of interest.
